# Epigenetic Regulation of the Epithelial to Mesenchymal Transition in Lung Cancer

**DOI:** 10.3390/cancers9070072

**Published:** 2017-06-24

**Authors:** Joëlle Roche, Robert M. Gemmill, Harry A. Drabkin

**Affiliations:** 1 Laboratoire Ecologie et Biologie des Interactions, Equipe SEVE, Université de Poitiers, UMR CNRS 7267, F-86073 Poitiers, France; 2 Division of Hematology-Oncology, Medical University of South Carolina, 39 Sabin St., MSC 635, Charleston, SC 29425, USA; gemmill@musc.edu (R.M.G.); drabkinh@gmail.com (H.A.D.)

**Keywords:** chromatin modifications, EMT, Epigenetics, Epitranscriptomics, EZH2, non-coding RNAs, NSCLC, SCLC

## Abstract

Lung cancer is the leading cause of cancer deaths worldwide. It is an aggressive and devastating cancer because of metastasis triggered by enhanced migration and invasion, and resistance to cytotoxic chemotherapy. The epithelial to mesenchymal transition (EMT) is a fundamental developmental process that is reactivated in wound healing and a variety of diseases including cancer where it promotes migration/invasion and metastasis, resistance to treatment, and generation and maintenance of cancer stem cells. The induction of EMT is associated with reprogramming of the epigenome. This review focuses on major mechanisms of epigenetic regulation mainly in lung cancer with recent data on EZH2 (enhancer of zeste 2 polycomb repressive complex 2 subunit ), the catalytic subunit of the PRC2 (Polycomb Group PcG), that behaves as an oncogene in lung cancer associated with gene repression, non-coding RNAs and the epitranscriptome.

## 1. Introduction

Lung cancer is the leading cause of cancer deaths worldwide [1]. Non-small cell lung cancer (NSCLC) accounts for about 85% of cases and includes adenocarcinomas, characterized by *RAS* or *EGFR* mutations, squamous cell carcinoma with *FGFR1* amplification, *PTEN* or *PIK3C*A mutations [2,3], and large cell carcinoma, which are highly heterogeneous. The recent WHO classification [4] now distributes large cell carcinoma into either NSCLC or small cell lung cancer (SCLC) depending on its characteristics. SCLC accounts for approximately 15% of lung tumors and is among the most aggressive tumor types because of high proliferation and early metastasis. It is observed almost exclusively in heavy smokers, and is characterized by gene inactivation of p53 (*TP53*) and retinoblastoma (*RB1*), along with expression of neuroendocrine markers [5,6,7]. Metastasis is triggered by enhanced cellular migration and invasion, and the epithelial to mesenchymal transition (EMT) described in the following paragraph, believed to play a role in this process.

**Figure 1:**
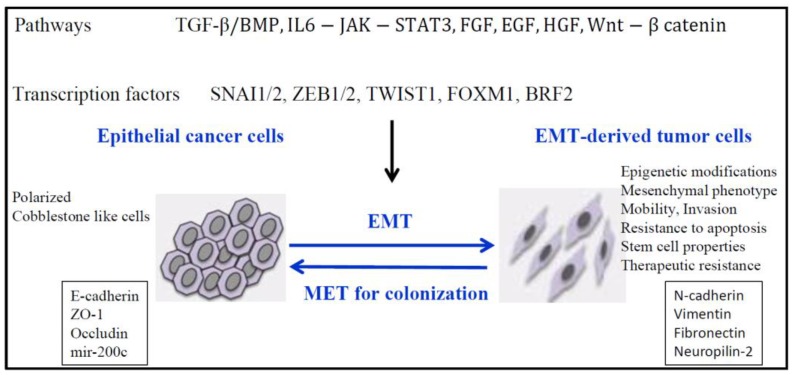
EMT (Epithelial to Mesenchymal transition ) and epigenetic modifications. EMT is induced by different pathways that involve different transcription factors necessary to repress epithelial genes and to activate mesenchymal genes.

EMT is a fundamental developmental process that is reactivated in wound healing and a variety of diseases including cancer where it induces the development of metastasis, resistance to treatment, and generation and maintenance of cancer stem cells [8,9,10,11,12,13]. However, two recent studies show that EMT can be dispensable for metastasis, but nevertheless contributed substantially to chemoresistance [14,15]. These studies were recently questioned because they coincided with a time when the definition of the EMT was undergoing re-evaluation because of possible multiple partial EMT states, and the proofs that EMT did not occur during metastasis were not completely supported [13] The EMT program includes loss of intercellular adhesion with loss of tight junctions, adherens and gap junctions, increased invasion and migration, and a switch from epithelial to mesenchymal gene expression patterns. E-cadherin and ZO-1 expression is often lost, while in contrast there is a gain of N-cadherin, Vimentin and Fibronectin expression (Figure 1). In TGFβ-induced EMT in lung cancer cells, we found increased expression of Neuropilin-2 (NRP2), the receptor for class 3 semaphorins, ligands providing guidance to and control of cell movement [16]. Most recently, we found that TGFβ preferentially induced NRP2b, an understudied isoform of NRP2 that was responsible for promoting the oncogenic response in cell lines and correlated with tumor progression in patients [17]. In contrast, the level of SEMA3F (semaphorin 3F), a secreted semaphorin with potent antitumor activity that binds NRP2, was decreased in a ZEB1-induced EMT lung cancer model [18]. Of interest, neuropilins bind TGFβ suggesting a function of NRPs in TGFβ response, and additional ligands such as VEGF (Vascular Endothelial Growth Factor), HGF (Hepatocyte Growth Factor), platelet-derived growth factor (PDGF), and EGF (Epidermal Growth Factor) [19].

Many EMT studies have focused mostly on cell-based experimental models and have focused on a small number of gene promoters that are epigenetically regulated, such as E-cadherin (*CDH1*) [20,21,22,23]. However, the direct involvement of EMT in tumor progression and metastatic spread has been questioned. In fact, the difficulties to characterize EMT in vivo come, in part, from the fact that EMT can be transient and partial. Indeed, EMT is increasingly viewed as a program generating cells with a spectrum of multiple states between epithelial and mesenchymal extremes, and partial EMT would be frequent in tumors [12,13]. Our previous study on 22 NSCLC cell lines showed that they are clearly in different states with more epithelial traits such as NCI-H358 cells or with more mesenchymal traits such as A549, NCI-H661, and NCI-H460 cells [24]. Indeed, a recent integrative approach combining mRNA, miRNA, DNA methylation, and proteomic profiles of 38 cell lines representative of lung adenocarcinoma heterogeneity, and functional profiles consisting of cell invasiveness, adhesion, and motility, defined cell lines as epithelial (E), mesenchymal (M), or intermediate/hybrid (E/M) with mixed epithelial and mesenchymal characteristics. Aggressive hybrid cell lines were characterized with a signature shared with mesenchymal cell lines: upregulation of cytoskeletal and actin-binding proteins [25]. Such E/M hybrid cells have been observed among circulating tumors cells and are associated with metastasis [26].

In lung cancer, TGFβ is one of the most important physiologic EMT inducers, along with other factors such as HGF, FGF (Fibroblast Growth Factor), IGF (Insulin-like Growth factor-1)/PDGF, EGF/VEGF, each secreted by the tumor or/and its microenvironment and acting through downstream pathways including Wnt/β-catenin, TGFβ/SMAD, Notch, MAPK/ERK, and PI3K/Akt [27,28].

The induction of EMT is associated with reprogramming of the epigenome by the action of transcription factors (such as TWIST, SNAIL, SLUG, and ZEB1/2), non-coding RNAs such as micro-RNAs (miRNAs) and long non-coding RNAs (lncRNAs). Here, we focus on epigenetic regulation mainly in lung cancer, including recent data on non-coding RNAs and the epitranscriptome.

## 2. Epigenetics

Epigenetic regulation is normally dynamic and reversible, affecting chromatin structure for the regulation of gene expression without modification of DNA sequences [29,30]. It includes the incorporation of histone variants, covalent histone modifications, nucleosome re-positioning, DNA methylation, changes in the expression of non-coding RNAs, and RNA post-translational modifications. The impact of epigenetic changes in cancer is reflected by altered gene expression, reactivation of endogenous retro-elements and genomic instability.

The nucleosome is the basic chromatin unit consisting of a protein core formed by two copies of histones H2A, H2B, H3 and H4, encircled by 180–200 bp of DNA stabilized by histone H1 that binds DNA at its entry and exit points of the nucleosome. Histones are post-translationally modified by phosphorylation, acetylation, methylation and ubiquitylation (among others). DNA can be covalently modified by methylation, usually on cytosine residues immediately preceding guanosine (CpG), without altering the sequence of base pairs. The epigenome refers to this collective set of DNA and histone modifications, along with their precise distribution along chromatin and the dynamic changes in this pattern mediated by regulatory processes. DNA methylation at CpG is one of the best studied epigenetic marks. DNA methylation and histone modifications are added by enzymes called “writers” and removed by “erasers”. These modifications are further recognized by proteins called “readers” that recruit various adapters for regulation of gene expression [31] (Table 1).

DNA can be methylated at the fifth position of cytosine (5mC) in the CpG dinucleotide context by DNA methyltransferases and further demethylated by TET enzymes with sequential oxidation of 5mC to 5-hydroxymethylcytosine (5hmC), 5-formylcytosine (5fC), and finally 5-carboxylcytosine (5caC). 5fC and 5caC are further cleaved by thymine-DNA glycosylases to restore unmethylated cytosine via the base-excision repair machinery [33]. In cancer, abnormal DNA methylation is observed with a global decrease of DNA methylation on repetitive sequences, and transposons, associated with chromosome anomalies and active transposition [34,35]. Of interest, gene bodies of tumor suppressor genes are undermethylated but, in contrast, their promoters are hypermethylated. Gene promoter hypermethylation is generally associated with transcriptional repression.

Histones are covalently modified primarily on their amino-terminal tails [36]. The nature and combination of histone modifications forms a “histone code” that affects transcription of nearby genes [37].

Whole genome and transcriptome sequencing established that about 70% of the genome is transcribed into non-coding RNAs. These non-coding RNAs emerged as a novel class of functional molecules with involvement in a variety of physiological processes and in tumorigenesis when misregulated. Non-coding RNAs are divided in two classes depending on their size: the small non-coding RNA group which is less than 200 nucleotides long includes microRNAs (miRNA), while the long non-coding RNA (lncRNA) group extends from 200 nucleotides to over 100 kb. More than 2000 human miRNAs have been identified and a large fraction is deregulated in cancers including lung cancer [38]. miRNAs regulate around 30% of coding genes at the post-transcriptional and translational levels and each typically targets multiple genes within a pathway. LncRNAs belong to a very heterogeneous group of transcripts (for review see [39]). Interestingly, the architecture of lncRNAs is complex: different structural domains can be combined by alternative splicing to sense or bind other RNAs, proteins and possibly DNA [40]. In addition, they undergo conformational switches that modulate their functions. Consequently, lncRNAs are considered as platforms to perform multiple functions in the cell and they act either in *cis* or in *trans*. They are involved in chromatin remodeling, transcriptional co-activation and co-repression, protein inhibition, post-transcriptional modifications (splicing), decoy binding platforms, mRNA stabilization, and more recently in nuclear organization of multichromosomal regions [41,42,43,44,45,46,47,48]. Among these functions, lncRNAs bind to chromatin-modifying proteins for their recruitment to specific sites in the genome, and lncRNAs act as competing endogenous RNAs (ceRNAs) for miRNAs. These two functions have been described in the epigenetic regulation of EMT [49,50,51].

To these classical epigenetic modifications, epitranscriptomics and the RNA code came into the spotlight recently [52]. Epigenetic modifications not only affect DNA and proteins, but also coding and non-coding RNAs. RNAs show a diverse spectrum of more than 100 modifications including *N*^7^-methylguanosine (m^7^G), *N*^6^-methyladenosine (m^6^A), m^5^C, pseudouridine, and queuosine [32,53]. These modifications are reversible, dynamic, and important for gene expression. Enzymes that are writers, erasers, and protein readers of these RNA modifications, analogous to the equivalent functions for histone modification, have been identified [54] (Table 1). However, additional technology development is necessary to detect, quantify, and map these modifications [52,55]. This is a challenging and exciting field still in its early stage, with much to be discovered to understand the role of RNA epigenetic modifications.

## 3. Epigenetics and Lung Cancer

Mutations in genes encoding epigenetic proteins and abnormalities in epigenetic regulation are clearly linked to cancer (for review see [56]). They have been described previously for lung cancer [34,57,58,59,60].

Large-scale genomic studies have identified recurrent alterations of epigenetic regulators in lung cancer (Table 2).

In SCLCs, mutations are found for histone acetyltransferases, histone methyltransferases and demethylases, and remodeling factors [7,62,66]. High frequency truncating mutations for the H3K4 histone methyltransferase *KMT2D/MLL2* gene have been reported in 17% of SCLC cell lines and 8% of SCLC tumors, and *KMTD2/MLL2* loss is associated with reduced H3K4me1 and impaired enhancer function [62]. Less frequent are mutations in the H3K27 histone demethylase, *KDM6A/UTX* gene, that occur in an exclusive fashion with *KMTD2/MLL2* mutations. Of interest, bivalent promoters are characterized by the presence of both H3K4me3 active mark and H3K27me3 inactive mark that poise developmental genes, enabling them to respond rapidly to suitable stimuli [67]. Therefore, *KMTD2/MLL2* or *KDM6A/UTX* mutations in addition to abnormal EZH2 expression (see below) lead to impaired expression of genes under control of these bivalent promoters.

For NSCLC, the most frequent mutations are found in the chromatin remodeling factors including SMARCA4/BRG1, and the H3K36 histone methyltransferase SETD2, in 6% and 9% of adenocarcinomas, respectively. BRG1 is one of the two ATPase subunits in the SWI/SNF chromatin-remodeling complex and has been reported to be frequently mutated or silenced in primary human NSCLC tumors and cell lines (for reviews [68,69]). To a lesser extent, the DOT1L methyltransferase for H3K79 is mutated in 3% of lung adenocarcinomas [64].

Abnormal expression of epigenetic players in lung cancer includes EZH2 overexpression in SCLC and NSCLC, where EZH2 acts as an oncogene in these tissues. EZH2 is the catalytic subunit of the PRC2 (Polycomb Group PcG) complex, which includes additional core components (SUZ12, EED, RBBP4). It mediates methylation of lysine 27 on histone H3 (H3K27), associated with gene repression. EZH2 overexpression in lung cancer is common and associated with aggressive tumor characteristics, advanced stage and poor prognosis [70,71,72,73]. In cell lines and patient-derived xenografts, EZH2 inhibition attenuated cell-cycle progression, growth and invasion [72,73,74,75]. Interestingly, tobacco smoke causes upregulation of Wnt signaling by recruiting EZH2 to suppress Dickkopf-1, a Wnt antagonist [76]. 

At the transcriptional level, EZH2 is suppressed by the pRB protein. At least in part, this explains the upregulation of EZH2 in SCLC, a disease with near-universal mutation of *RB* [77,78]. Of interest, the retinoblastoma protein occupies repetitive sequences that include tandem sequence repeats and interspersed repeats (endogenous retroviruses and LINE-1 elements) in somatic cells and is associated with EZH2. Consequently, H3K27 trimethylation maintains silencing of these sequences [79]. In cancer cells, these repetitive sequences can be reactivated. When pRB binding to these sequences is inhibited, the consequence is an increased susceptibility for spontaneous lymphoma in mice. Therefore, loss of pRB in SCLC or abnormal level of EZH2 would be involved in repetitive sequence expression responsible for mutagenesis and multiple possible aberrations.

Links between epitranscriptome and cancer have emerged with the discovery that FTO (Fat mass and obesity-associated protein), first identified as the m^6^A eraser, but recently described as the m^6^Am (*N*^6^, 2′-*O*-dimethyladenosine) eraser, is abnormally expressed in acute myeloid leukemia [80], and affects the stability and subcellular location of mRNAs [81]. FTO is also overexpressed in breast cancer compared to adjacent breast tissues [82] and overexpression is associated with higher metastatic potential and resistance to chemotherapy in an in vitro cellular breast cancer model [83]. The ncRNA epitranscriptome is also altered in cancer [32] but more data are needed for lung cancer.

## 4. EMT and Epigenetics in Lung Cancer

A review about epigenetic regulation of EMT in NSCLC was recently published [28]. Additional data will be described below for lung cancer.

The involvement of the Snail and ZEB1 transcription factors strongly supports epigenetic modifications during EMT. Indeed, Snail has been described as a pseudosubstrate “hook” for the histone demethylase LSD1 that demethylates H3K4 [84,85] and H3K9 (Table 1). Through its SNAG domain that mimics a histone H3 tail, Snail binds to LSD1 and the complex is stabilized by association of the co-repressor CoREST. HDAC1/2 and PRC2 are later recruited for *CDH1* repression in cancer cells, including mammary epithelial tumor cells. SNAIL was also found to associate with G9a (EHMT2), a major histone methyltransferase responsible for creating the H3K9me2 repressive mark [86] and which also contributes to methylation of H3K27 [87] (Table 1). In addition, SNAIL interacts with SUV39H1 (another histone methyltransferase) during EMT induced by TGF-β and mediates silencing of *CDH1* by the addition of a third methyl group to H3K9 that confers a more stable and durable repressive state than H3K9me2 [88].

ZEB1 also associates with partners involved in epigenetic regulation. For gene activation, ZEB1 associates with the histone acetyltransferases (HATs) p300, PCAF, and Tip60. In contrast, as a repressor, ZEB1 interacts with CtBP [89] and recruits class I and II histone deacetylases (HDACs) [90]. Of interest, CtBP associates with several other partners including the Polycomb complex PRC2 as described above, G9a (EHMT2), the co-repressor CoREST, and the histone demethylase LSD1 (for review see [91,92]). ZEB1 can also recruit the nicotinamide adenine dinucleotide-dependent sirtuin, SIRT1, in prostate cancer cells to repress *CDH1* and to induce several EMT markers [93]. In addition, ZEB1 interacts via its N-terminal region with BRG1 to repress *CDH1* in colon cancer cells [94].

**Figure 2:**
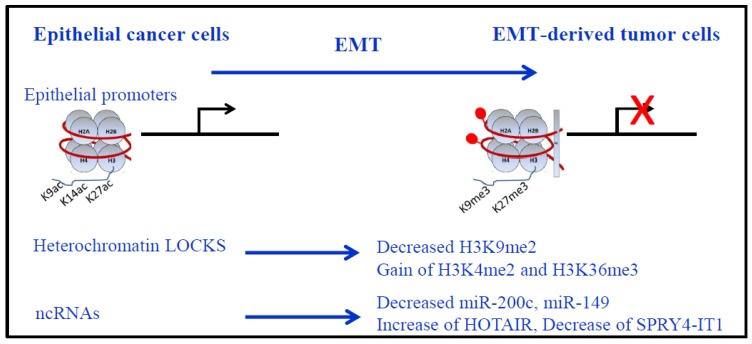
Epigenetic modifications during EMT. Nucleosomes are represented with DNA in red, wrapped around the histone core (2 copies of each histone H2A, H2B, H3 and H4) with the presence of histone H1 in more compact chromatin. Repression of epithelial genes (red cross) is shown with corresponding epigenetic modifications on their promoters: more compact chromatin, decreased H3K9/14/27ac and gain of H3K9me3 and H3K27me3, associated to DNA methylation (red lollipop). Of note, only major modifications for histone H3 are presented. LOCKS (long-range chromatin domains) epigenetic marks during EMT: Global reduction in the heterochromatin mark H3K9me2, increase in the euchromatin mark H3K4me3, and increase in the transcriptional mark H3K36me3. Non-coding RNAs (ncRNAs) expressions are also modified during EMT for miRNAs (loss of miR-200c and miR-149) and lncRNAs (increased HOTAIR and decreased SPRYA-IT1 in lung cancer).

In H358 NSCLC cells where EMT was induced by ZEB1 expression, we used Western blot and immunocytochemistry to identify a global decrease in H3K27 acetylation. We found that ZEB1 binding resulted in decreased acetylation of histone H3 on residues K9 and K27 of target genes [95] (Figure 2). Our results showed that ZEB1 increased trimethylation of H3K27 on selected target genes and that H3K4me2 did not change drastically upon ZEB1 binding. These results suggested that ZEB1 recruitment of PRC2 during EMT would create bivalent domains for epithelial gene repression. Such genes would then be poised for rapid reversal of the repressed state to facilitate the mesenchymal to epithelial transition (MET) thought to be important for the subsequent growth of metastatic deposits. This hypothesis fits with the PRC2 repressive activity on promoters with the activating mark H3K4me2/3 [96]. 

Recent studies showed that specific “long-range” chromatin domains across the genome, called “LOCKs”, found in non-repetitive heterochromatin domains up to several megabases, are epigenetically remodeled as a major driving force during EMT [97]. A global reduction in the heterochromatin mark H3K9me2, an increase in the euchromatin mark, H3K4me3, and an increase in the transcriptional mark, H3K36me3, were described (Figure 2). These changes depended largely on LSD1. Of interest, DNA methylation was preserved across the genome during EMT.

Epigenetic modifications also affect super-enhancers that are localized to unique relatively small subsets of genes that differ between cell states during EMT. They are often found at key oncogenes, such as *MYC.* Loss of BRD4, a bromodomain protein that binds acetylated histones, or its pharmacological inhibition, can cause super enhancer-mediated gene expression to be lost [98]. In SCLCs, MYCL, one of the three MYC family oncogenes, is often overexpressed and treatment with JQ1, a BET bromodomain inhibitor, considerably decreased cell growth, induced cell cycle arrest and apoptosis, and reduced expression of the three *MYC* genes [99].

Non-coding RNAs contribute to EMT in NSCLC. For example, micro-RNAs including miR-132 and miR-149 that target ZEB2 and FOXM1 (Forkhead box M1), respectively, and downregulation of miR-149 was inversely correlated with invasive and EMT phenotypes in NSCLC [100]. The miR-200 family (that includes miR-200a, miR-200b, miR-200c, miR-141, and miR-429) received a lot of interest because of a negative transcriptional feedback loop between miR-200c and ZEB1 [101,102]. In our ZEB1-induced EMT model in the H358 NSCLC cell line, we observed a decrease of miR-200c during EMT [95]. Moreover, in the HCC4006 NSCLC cell line that became resistant to EGFR inhibitors, acquired resistance was associated with ZEB1, an EMT phenotype, and decreased miR-200c levels [103].

Several long non-coding RNAs (lncRNAs) are also associated with an EMT signature and aggressiveness [51,60]. In NSCLC, a positive correlation with lymph node metastasis was described for HOTAIR, CARLO-5, PVT1, MVIH and ZXF1, while a negative correlation was found with MEG3, SPRY4-ITI, BANCR and GAS6-AS1 [60]. Among these lncRNAs, HOTAIR and SPRY4-ITI are of interest in EMT regulation (Figure 2). HOTAIR interacts with the PRC2 complex to induce in *trans* H3K27 trimethylation of the *HOXD* locus, and the LSD1/coREST/REST complex that catalyzes H3K4me2 demethylation. However, this model of PRC2 binding to HOTAIR for targeting specific sequences was recently challenged, since PRC2 interacts with strong affinity but weak specificity to a wide set of all RNAs. Indeed, PRC2 is dispensable for HOTAIR-mediated transcriptional repression while PRC2 recruitment and H3K27 trimethylation were proposed to occur due to HOTAIR-dependent silencing [104,105]. How this happens mechanistically will require further investigation. HOTAIR is also involved in the maintenance of stemness and EMT in colon and breast cancer cell lines [106]. SPRY4-ITI is an inhibitor of the MAPK signaling pathway, and tumor suppressive functions were described in NSCLC cell lines. SPRY4-ITI inhibits NSCLC cell migration/invasion, and suppresses metastasis. SPRY4-ITI is downregulated by EZH2 and is involved in the modulation of EMT through induction of E-cadherin expression and repression of vimentin [107].

MALAT-1 (metastasis associated lung adenocarcinoma transcript-1, also called NEAT2) is a predictive marker for metastasis and shorter survival in early stage lung adenocarcinoma [51,60]. It can act as a ceRNA for several miRNAs including miR-200c, and can recruit PRC2 subunits (EZH2 and Suz12) to the *CDH1* promoter. CCAT2 (colon cancer associated transcript 2) is also a predictive marker for metastasis in lung cancer. In addition, lncRNAs are associated with EMT, stem cell properties and drug resistance. For example, UCA1, BC087858 and GAS5 are associated with resistance to EGFR tyrosine kinase inhibitors (EGFR-TKIs) in lung cancer possibly through the Akt signaling pathway.

Lastly, the epitranscriptome is likely involved in EMT but to our knowledge data are missing in lung cancer. However, we suspect that modifications of lncRNAs MALAT1 and TUG1 are candidates. Indeed, for MALAT1, two major m^6^A sites were found at consensus sites in two hairpin stem structures in several human cell lines including, MDA-MB231 breast cancer cells [108]. The consequence of this modification would destabilize these secondary structures and would modify interactions with RNA binding proteins. One can speculate that FTO overexpression would alter m^6^A levels during EMT. The other lncRNA TUG1 function might be altered as well, as one site shows m^6^A modification [108].

## 5. Therapeutic Inhibition of EMT

From a therapeutic standpoint, epigenetic processes are targets for therapeutic exploitation [11,22,31], and epigenetic regulators implicated in cancer and their corresponding inhibitors are described in recent reviews [56,109]. Some of them would be appropriate to target EMT in lung cancer. EZH2 inhibitors would be good candidates because of EZH2 oncogenic status in lung cancer, and some are in clinical development [110]. We also suspect that bromodomain inhibitors such as JQ1 (known as TEN-010 in clinical trials), the BET inhibitor for BRD4, would be appropriate because JQ1 demonstrated efficacy in blocking tumor progression in several cancer models including SCLC lung cancer [98,99]. One possible mechanism would be a decrease of C-Myc recruitment to EZH2 and consequently reduced EZH2 expression, as shown in bladder cancer [111]. Combined treatments with histone deacetylase inhibitors (such as SAHA, i.e., vorinostat, an FDA-approved drug) and bromodomain inhibitors should be considered in NSCLC for their synergistic therapeutic benefit seen in animal models [112,113]. HDAC inhibitors were also shown to induce E-cadherin in lung cancer and this restoration increased sensitivity to EGFR inhibitors [114,115]. Although the mode of action of the two drugs is multifaceted, one reason for this benefit would be their immunostimulatory effects on tumor growth arrest and prolonged survival as shown in a mouse model for lung adenocarcinoma [113].

## 6. Conclusions

In this review, epigenetics regulation of EMT was described in lung cancer with a particular interest in EZH2, an oncogene in lung tumors. Recent data on non-coding RNAs and epitranscriptomics were introduced. Attempts to inhibit EMT should consider combined treatments with HDAC and bromodomain inhibitors. However, caution is needed with these treatments to address the mesenchymal to epithelial transition (MET) that occurs at sites of metastasis.

## Figures and Tables

**Table 1:** Epigenetic modifications. Only DNA and RNA methylation, histone acetylation and some epigenetic modifications for histone H3 are shown with the corresponding players (writers, erasers and readers). For H3K79 methylation, DOT1L functions are multiple: it is involved in telomeric silencing, cellular development, cell-cycle checkpoint, DNA repair, and regulation of transcription. For RNA, only m^5^C and m^6^A are described but other modifications can be found in the review by Esteller and Pandolfi (2017) [32]. ** G9a weakly methylates H3K27.

	Epigenetic Modification		Function	Writer	Eraser	Reader
**DNA**	CpG Methylation		Transcriptional repression	DNMT1/3A/3B	TET1/2/3	MeCP, MBD1-4, UHRF1
**RNA**	m^5^C		tRNA stabilization, translation, immune response	DNMT2 (=TRDMT1) NSUN family	TET	not identified
	m^6^A		RNA splicing, export, stability, immune tolerance	METTL3/4, WTAP	FTO, ALKBH5	YTHD family, HuR HNRNPA2B1
**Histones**	Lysine Acetylation		Transcriptional activation	HAT	HDAC1-11, SIRT1-7	BRD bromodomain
	Lysine Methylation	HH3K4	Transcriptional activation	MLL1-5, SET1A/B, SET7/9, ASH1L	LSD1, JARID1a/b	Chromodomain, Tudor, MBT repeat, PHD finger
		HH3K9	Transcriptional repression	G9a(EHMT2) SUV39H1/2	LSD1, GASC1	
		HH3K27	Transcriptional repression	EZH1/2, G9a **	UTX, JMJD3	
		HH3K36	Transcriptional activation	SETD2, ASH1L, ASF1A, NSD1-3, SMYD2	Rph1/KDM4 Jhdm1b/Kdm2b	
		HH3K79	Transcriptional regulation	DOT1L, RE-IIBP	not known	

**Table 2:** Mutations in epigenetic regulators in lung cancers. ADC: Adenocarcinoma; SC: Squamous cell carcinoma.

Lung Cancer	Gene	Function	Mutation	References
**SCLC**	*KAT3A/CREBBP*	histone acetytransferase	inactivating mutation	[7]
	*KAT3B/EP300*	histone acetytransferase	inactivating mutation	[7]
	*KAT6B*	H3K23 histone acetytransferase	genomic loss	[61]
	*KMT2D/MLL2*	H3K4me1/2 histone methyltransferase	frequent inactivation	[7,62]
	*KDM6A/UTX*	H3K27 histone demethylase	truncating mutation in a small number of SCLC patients	[62,63]
	*PBRM1*	chromatin remodeling factor	mutation	[62]
	*ARID1A*		mutation	[62]
	*ARID1B*		mutation	[62]
**NSCLC**	*KMT2D/MLL2*	H3K4me1/2 histone methyltransferase	mutation in 20% SC	[3]
	*SETD2*	H3K36 histone methyltransferase	9% ADC	[3]
	*DOT1L*	H3K79 histone methyltransferase	3% ADC	[64]
	*ARID1A*	chromatin remodeling factor	7% ADC	[3]
	*ARID1B*		6% ADC	
	*ARID2*		7% ADC	
	*SMARCA4/BRG1*		6% ADC	
	*BRD3*	Bromodomain, binds hyperacetylated chromatin		[65]

## Abbreviations

The following abbreviations are used in this manuscript:

ADC: Adenocarcinoma
ceRNA: competing endogenous RNA
EGF: Epidermal Growth Factor
EGFR: Epidermal Growth Factor Recptor
EMT: Epithelial to Mesenchymal transition
EZH2: enhancer of zeste 2 polycomb repressive complex 2 subunit
HDAC: Histone deacetylase
HGF: Hepatocyte Growth Factor
lncRNA: long non-coding RNA
MET: Mesenchymal to Epithelial Transition
NSCLC: noRsmall cell lung cancer
PRC2: polycomb repressive complex 2
SC: squamous cell carcinoma
SCLC: small cell lung cancer
VEGF: Vascular Endothelial Growth Factor
